# Hippocampal Remapping Is Constrained by Sparseness rather than Capacity

**DOI:** 10.1371/journal.pcbi.1003986

**Published:** 2014-12-04

**Authors:** Axel Kammerer, Christian Leibold

**Affiliations:** 1 Department Biologie II, Ludwig-Maximilians-Universität München, Planegg, Germany; 2 Graduate School for Systemic Neurosciences, Ludwig-Maximilians-Universität München, Planegg, Germany; University of Tübingen and Max Planck Institute for Biologial Cybernetics, Germany

## Abstract

Grid cells in the medial entorhinal cortex encode space with firing fields that are arranged on the nodes of spatial hexagonal lattices. Potential candidates to read out the space information of this grid code and to combine it with other sensory cues are hippocampal place cells. In this paper, we investigate a population of grid cells providing feed-forward input to place cells. The capacity of the underlying synaptic transformation is determined by both spatial acuity and the number of different spatial environments that can be represented. The codes for different environments arise from phase shifts of the periodical entorhinal cortex patterns that induce a global remapping of hippocampal place fields, i.e., a new random assignment of place fields for each environment. If only a single environment is encoded, the grid code can be read out at high acuity with only few place cells. A surplus in place cells can be used to store a space code for more environments via remapping. The number of stored environments can be increased even more efficiently by stronger recurrent inhibition and by partitioning the place cell population such that learning affects only a small fraction of them in each environment. We find that the spatial decoding acuity is much more resilient to multiple remappings than the sparseness of the place code. Since the hippocampal place code is sparse, we thus conclude that the projection from grid cells to the place cells is not using its full capacity to transfer space information. Both populations may encode different aspects of space.

## Author Summary

The mammalian brain represents space in the population of hippocampal place cells as well as in the population of medial entorhinal cortex grid cells. Since both populations are active at the same time, space information has to be synchronized between the two. Both brain areas are reciprocally connected, and it is unclear how the two codes influence each other. In this paper, we analyze a theoretical model of how a place code processes inputs from the grid cell population. The model shows that the sparseness of the place code poses a much stronger constraint than maximal information transfer. We thus conclude that the potentially high spatial acuity of the grid code cannot be efficiently conveyed to a sparse place cell population and thus propose that sparseness and spatial acuity are two independent objectives of the neuronal place representation.

## Introduction

The neuronal representation of space that is necessary for navigation and orientation has been traditionally assigned to the hippocampal place cell system [1], where cells fire only at few distinct locations and are silent elsewhere. Since the discovery of grid cells in the medial entorhinal cortex (MEC) [2], [3], which fire on a hexagonal spatial lattice, a second space representation is now known and it has become unclear what the functional differences of the two are. It is speculated that the MEC grid cells are predominantly used in path integration, whereas the place cells may connect position and context information [4]. From the coding perspective it is remarkable that the hippocampal place fields are considerably sparse, whereas the grid fields generate a much denser code with approximately one third of all grid cells active at any one time [3]. Since both networks are reciprocally connected anatomically [5], [6] and functionally [7], [8], the two space representations have to be synchronized. Understanding the interplay of both codes thus leads to the more general question of how a dense neuronal code can be efficiently transferred into a sparse code and vice versa.

In this paper, we focus on the mapping from grid to place cells. This extends previous coding approaches in so far as they studied the isolated grid cell system from a mainly information theoretic perspective [9], [10]. Here, we discuss a coding theory by including the further constraint that the grid code has to be readable by the place code at a similar and behaviorally relevant resolution, since we assume that space information is only relevant for the brain if it can be read out by other neurons. Employing two population models, for grid cells and place cells, we show that a relevant resolution of the order of centimeters can be easily transferred from a relatively small grid-cell to a relatively small place-cell population. Larger numbers (particularly of place cells) can thus be used to encode multiple environments [11] at a similar spatial resolution. Our model also shows that may interference owing to multiple environments reduces the sparseness of the hippocampal code much faster than it reduces the space information of the population patterns measured by the number of different environments that can be encoded at a given spatial resolution. These findings argue against a pure feed-forward model of place field formation from grid cells, consistent with recent experimental findings [7], [12]–[16].

## Results

**Figure 1:**
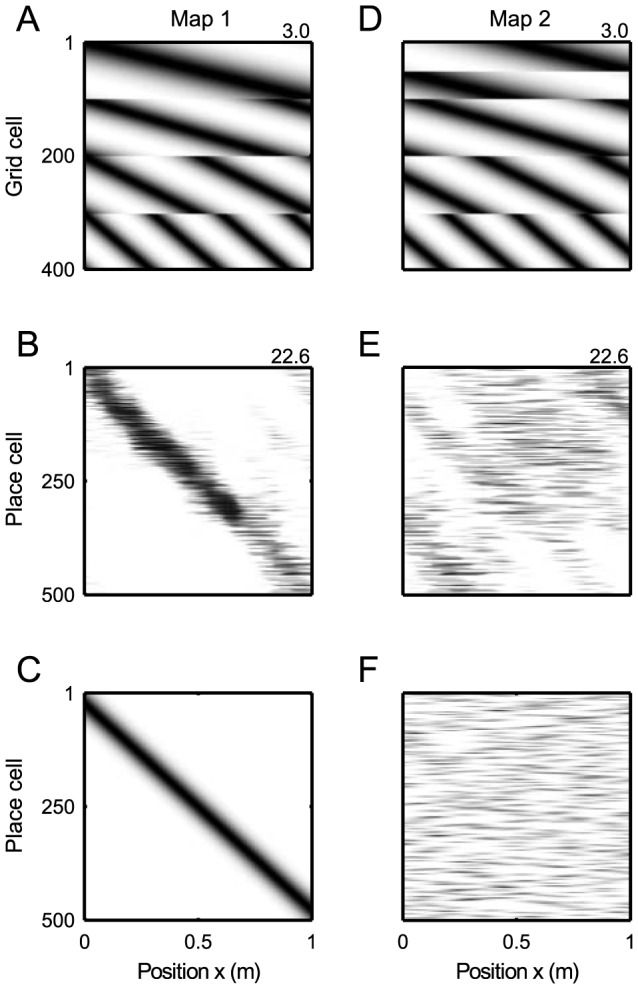
Hebbian learning of multiple linear tracks. (A) Grid cell firing maps for 400 grid cells with width constant 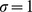 on a 1 meter linear track (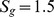, 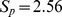, 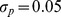 m, 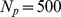). The cells are organized in 4 modules, with a period ratio of 1.67 to achieve a spatial period of 30 cm in the lowest module. The numbers at top right corners indicate the maximal spike count 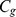 as a proxy for peak firing rate (see Materials and Methods). (B) Firing rates of place cells which received the grid field activity from A as an input. The corresponding synaptic connections were obtained from an Hebbian outer product rule based on the rate maps of the grid population in A and the ideal place field population (C). (D) To represent a second environment, the grid code from A is shifted by module-specific phases. (E) Globally remapped place code that is learned from the remapped rate maps in D and F. (F) Ideal place code in the second environment.

**Figure 2:**
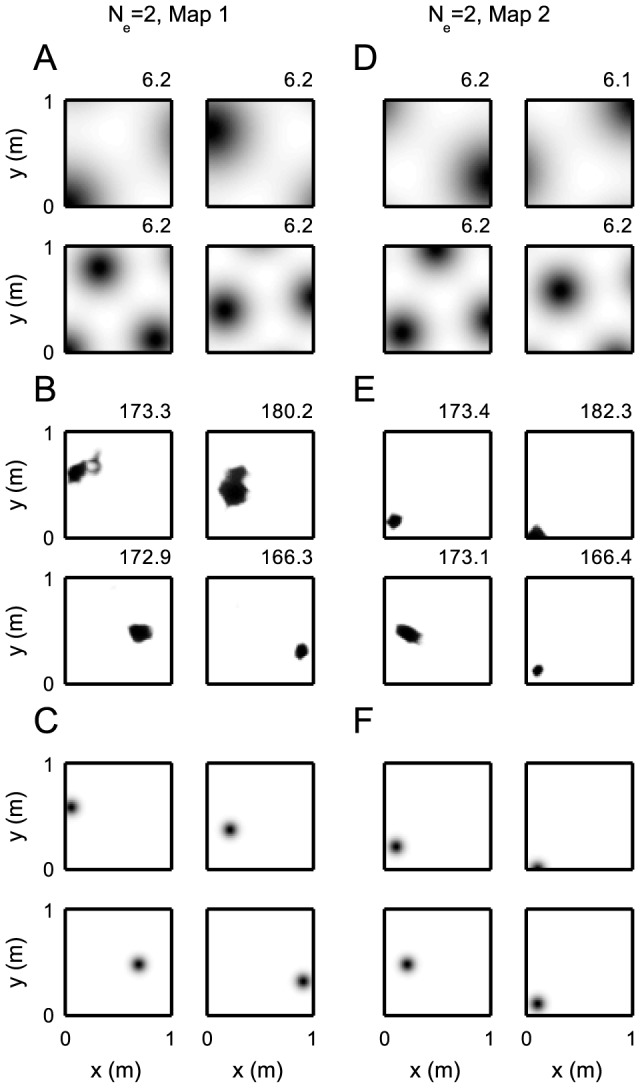
Two-dimensional rate maps for grid cells and place fields in two environments (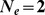). (A, D) Grid rates differ by module-specific phase shifts. Four example cells are shown, two from the first module (top) and two from the second (bottom). A total of four modules was used. Maximum spike counts 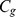 shown above each plot. (B, E) place cell rate maps for both remappings. Positions of place fields are set by Hebbian learning. (C, F) Desired place fields as used for Hebbian learning. Firing fields in C are distributed in a square lattice equidistantly across the environment. Fields in F are obtained by shuffling cell identities from C, which ensures equal coverage. Parameters are 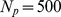 place cells and 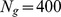 grid cells and 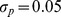 m, 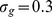, 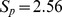, 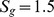. All other parameters are as for the one-dimensional case.

Here we briefly summarize the general structure of our model, whereas a detailed account is provided in the Materials and Methods Section. A population of 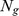 grid cells is connected to 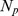 place cells via a feed-forward synaptic matrix. The grid cells are organized in four modules that differ in the spatial period (or grid spacing) of the periodic hexagonal firing patterns [17]. The neuronal activities of the MEC and hippocampal populations are assumed to encode either linear tracks or square boxes both of length 1 m (Figs. 1 and 2). Different environments are represented by phase shifts of the grid fields that are identical for all cells in a module [18] but random between modules [19].

The spike count of the grid cells is assumed to follow Poisson statistics. For the place cells we first define place fields that optimally cover the whole environment but are only used as teacher patterns in a training step in which we construct synaptic weights between grid cells and place cells by supervised Hebbian learning. The teacher place fields are randomly assigned in each environment (shuffling of place cells) resembling the global remapping [20] of hippocampal place fields found in experiments. For each such remapping synaptic weights are incremented according to the Hebb rule such that all shifted grid patterns activate the corresponding remapped place code.

Realizations of grid field spikes are projected via the learned feed-forward connections to the place field population that employs a soft winner-take-all mechanism (E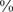-MAX rule) to emulate recurrent inhibition [21]. The activity from these simulations determines the actual firing fields and spike statistics of the place cells. The spatial acuity of both codes is measured by the empirical minimum mean square decoding error of single trial activity. The simulations are evaluated by a variety of measures including sparseness and the similarity between the place fields used during training and those obtained in the simulation.

The capacity of a spatial code consists of two components. First, the spatial resolution [9], or how precisely one can infer a spatial position. Second, how many different environments can be represented. Since different environments are obtained by MEC phase shifts and hippocampal remapping, all spatial information is conveyed by the same synaptic connections. Thus the multiple stored environments interfere at the cost of spatial resolution.

### Resolution of the grid code

**Figure 3:**
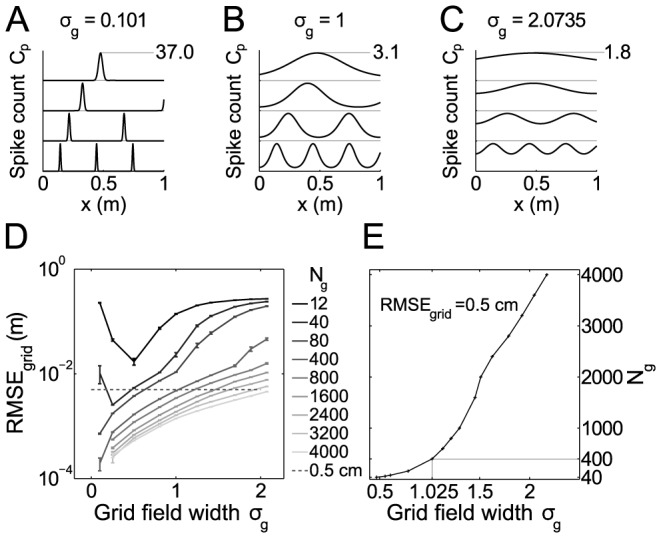
Root mean square error of grid cells (RMSE_grid_) on the linear track. (A–C) Example tuning curves for 4 cells from different modules and three choices of width constant 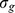. (D) RMSE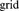 as a function of cell number 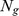 and tuning width 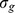. (E) Scaling of 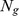 with 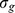 for fixed RMSE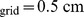. Parameters are 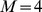, 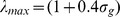 m, 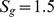.

To assess the ground truth of our model, we first evaluate the coding capacity of the grid cell population on a one-dimensional linear track (Fig. 3). The spatial resolution (denoted as root-mean square estimation error; RMSE) non-trivially depends on the tuning width 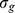 of the grid code and the number 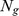 of neurons [9], [22]. Three examples of grid codes are shown in Fig. 3A–C for three different values of 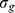. Grids as usually observed in MEC are most similar to the situation in Fig. 3B, whereas Fig. 3A and C illustrate settings with extremely thin and broad tuning curves, respectively. Thus, the biological value of 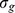 is about 1, which corresponds to a ratio between tuning width and spatial period of about 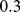 (see Fig. S4 of [3]). However, the RMSE non-monotonically depends on 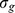 [22] with a minimum at rather thin tuning curves (Fig. 3D).

The resolution (RMSE) improves with 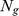 such that even for moderate cell numbers (several hundreds) it is easy to obtain spatial resolutions in the range of 1 mm and below. From a behavioral perspective, however, one may ask whether such a resolution is actually psychophysically reasonable, or even useful. We thus suggest that resolution is probably not the major objective of the grid code and test the alternative possibility that the grid code may be designed to display a reasonable spatial resolution in as many environments as possible. As a lower bound for such a reasonable resolution we postulate an RMSE of 0.5 cm (dashed line in Fig. 3D) and ask the question, which parameter setting in 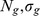-space would actually result in this behaviorally relevant RMSE (Fig. 3E). The minimum 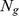 scales supra-linearly with 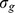, i.e. it flattens out for smaller 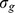. We thus argue that 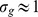 is a good choice because it still is in the super-linear regime requiring only relatively small cell numbers and at the same time results in tuning widths that are similar to biology (like Fig. 3B). For further analysis we thus fix the grid code to 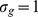 and 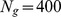.

### Resolution of the place code in a single environment

**Figure 4:**
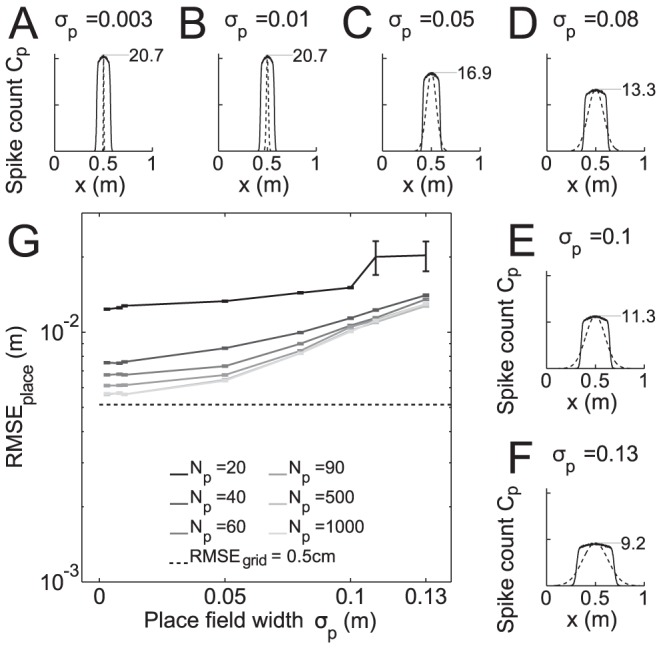
Root mean square error of place cells RMSE_place_ on linear track. (A–F) Place cell tuning functions (spike counts 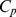 as a function of space 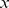). Dashed lines: teacher tuning curves used for training. Solid lines: tuning curves after learning averaged over 800 trials (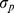 is the width of the teacher curves). (G) Place cell resolution RMSE_place_ as function of 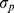 and 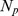. Grid cell resolution is shown as dashed line. Parameters used were 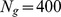, 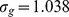, 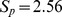, other parameters were as in Fig. 3.

The spatial acuity of the population code of grid cells can only be made use of if it can be read out by downstream centers. We therefore asked under which conditions the resolution of grid cell network from the previous subsection can be preserved in the place cell network under the ideal conditions that only one environment has to be represented (number of environments 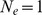); Fig. 4.

Since the tuning curves are actually learned there exists a clear lower bound for the tuning widths that reflects the minimal width of the grid cell population (Fig. 4A–F). Narrower place fields cannot be achieved by the present model even if the fields used during training are much narrower than the smallest grid fields. Similar as for the grid cell code, a reduction in the place field width effectively improves the RMSE, however, the resolution is limited by that of the grid code (0.5 cm). Therefore an increase in the number 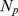 of place cells reduces the RMSE and the performance quickly converges to the minimum for 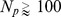; Fig. 4G. Only relatively few neurons are needed to achieve such a behaviorally relevant resolution, and thus we next asked how many different environments can be represented at this resolution.

### Multiple environments

**Figure 5:**
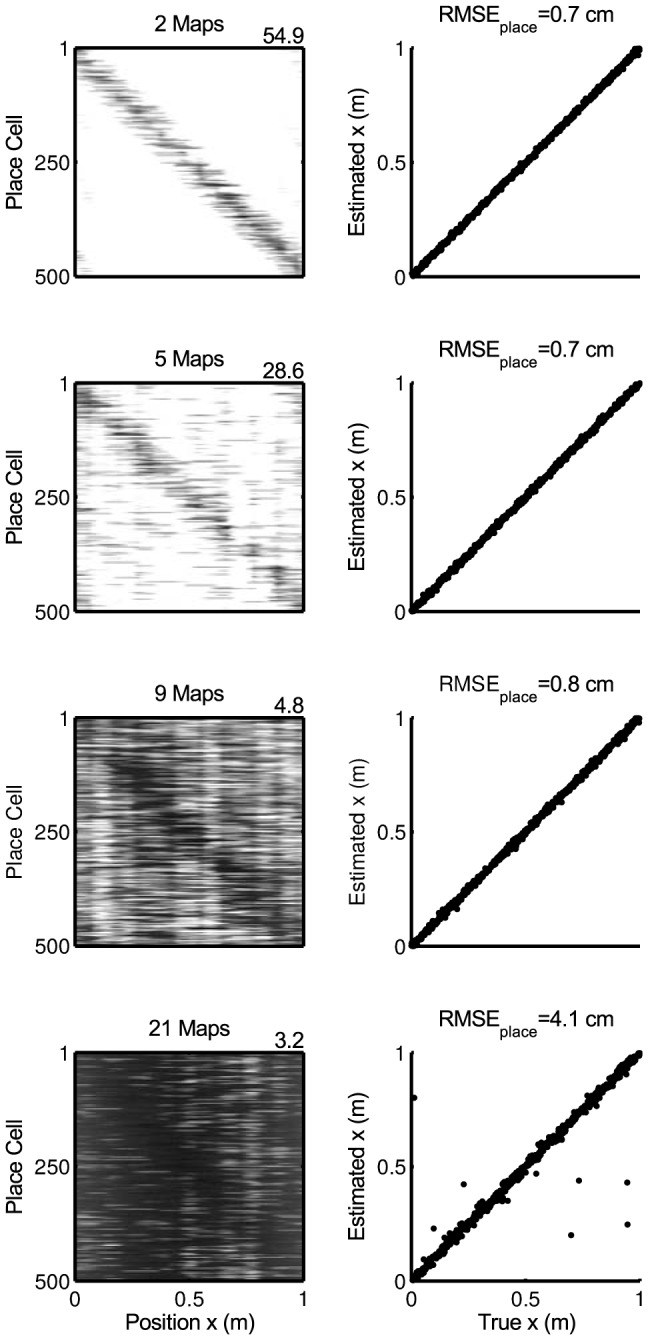
Quality of 1-d place code for increasing number of maps in a network with 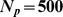 place cells and 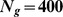 grid cells and 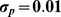 m, 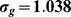, 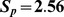, 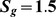. Left column: Rate map for environment 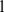. Right column: Position estimates from the place code as a function of real position.

**Figure 6:**
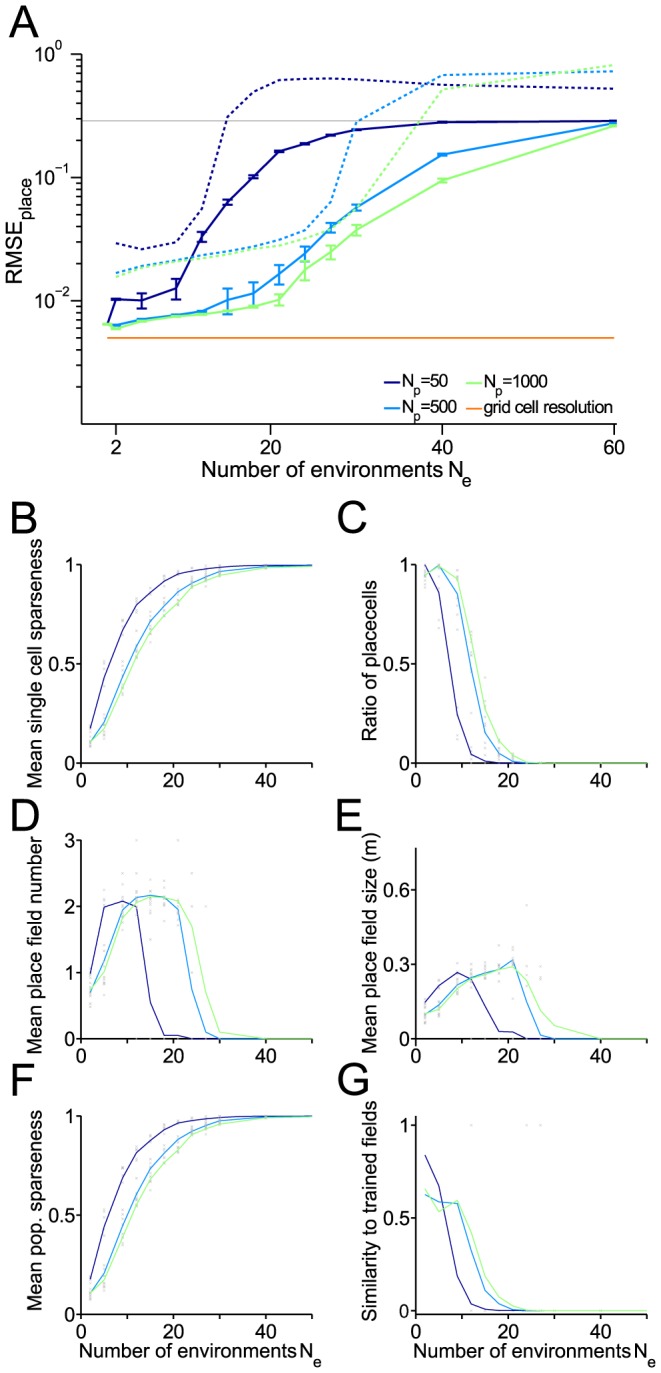
Capacity for storing remappings on the linear track. Place cell resolution and further measures as functions of the number 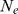 of remappings stored. (A) Root mean square error (RMSE) of place cells. Blue and green solid lines: Mean over realizations. Dashed lines: 99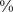 quantiles. Red line RMSE of the grid cell input. (B) Mean single cell sparseness. (C) Ratio of proper place cells. (D) Mean number of place fields for proper place cells. (E) Mean size of place fields for proper place cells. (F) Mean population sparseness. (G) Ratio of cells for which Hebbian learning was successful (according to the three similarity criteria defined in the Materials and Methods section). Parameters were 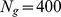, 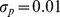 m, 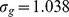, 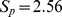, 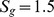, 4 modules, 20 realizations.

Storing multiple environments generates interferences of the place codes since each remapping taxes synaptic resources. Thus the spatial resolution of the place code is getting worse when storing multiple environments (Fig. 5). However, even for 21 remappings in our parameter regime (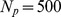) the decoding error is still relatively low (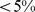). Also the number 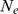 of remapped environments for which decoding is possible increases with the number of place cells (Fig. 6A), such that even for moderate place cell numbers 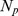 many environments can be easily decoded at physiological resolution.

Although space information is retained for considerably large values of 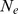, the place code degenerates already for much smaller 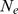. This degeneration is best described by a loss of sparseness (Fig. 6B, [23]) resulting from less localized firing fields, while the average spike count 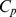 remains constant (see Materials and Methods). This delocalization results in a reduction of the number of proper place cells (Fig. 6C) which exhibit an increased number of regular-sized firing fields (Fig. 6D, E) before they cease to be place cells and are active over almost the whole track as indicated by a mean population sparseness (average fraction of active cells at a position) close to 1 (Fig. 6F). Also the firing fields quickly loose their similarity to the trained firing fields (Fig. 6G). From these observations we conclude that although a large number 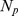 of putative place cells allow to reliably decode a large number of environments by remapping, the place field quality (i.e. the sparseness) of the encoding neurons disappears. Thus the observation of a sparse place code in the hippocampus must result from further objectives beyond decoding quality and remapping capacity.

### Generalization to open fields

**Figure 7:**
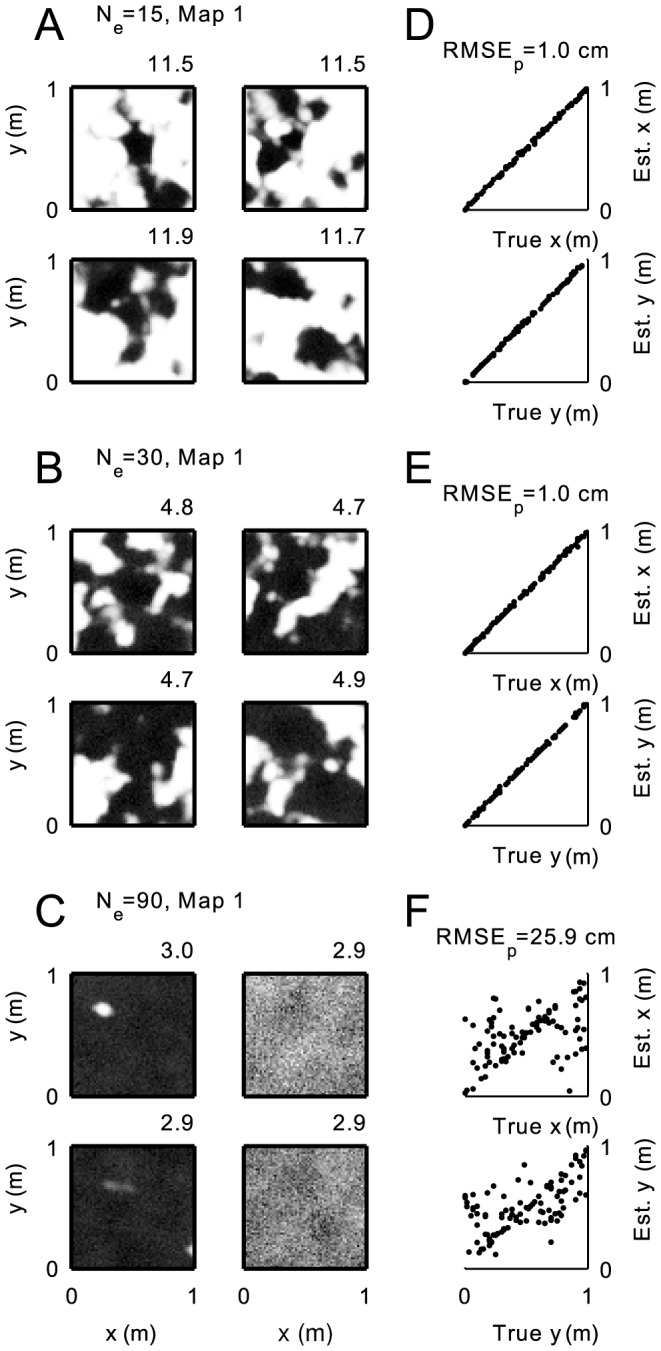
Quality of 2-d place code, for increasing number of stored environments 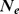. (A–C) Rate maps of four example cells for 15, 30, and 90 stored remappings. The desired place field positions (not shown) are identical to Fig. 2 C, but in this case are hardly achieved. (D–F): Minimum mean squared error estimates of position plotted against true position for 500 trials, again for 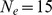, 30 and 90. Parameters as in Fig. 2.

**Figure 8:**
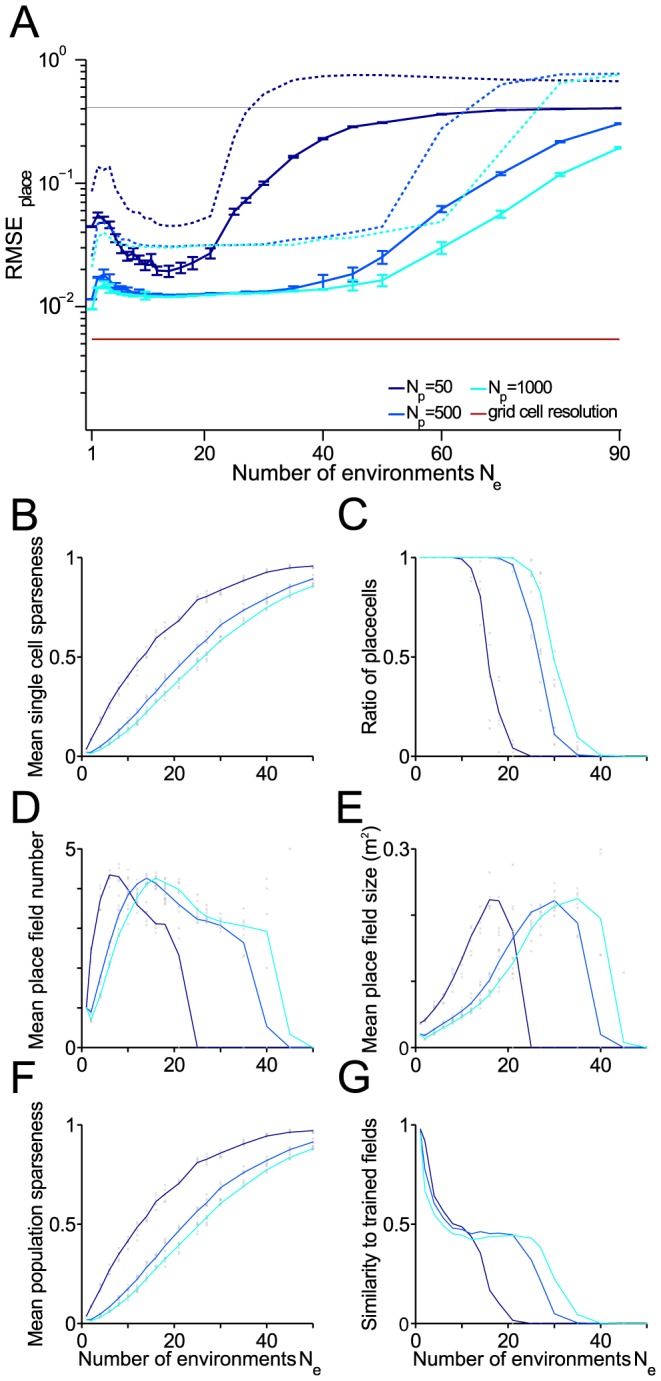
Capacity for storing remappings in a square box. Place cell resolution and further measures as functions of the number 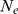 of remappings stored. (A) Root mean square error (RMSE) of place cells. Blue and green solid lines: Mean over realizations. Dashed lines: 99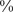 quantiles. Red line RMSE of the grid cell input. (B) Mean single cell sparseness. (C) Ratio of proper place cells. (D) Mean number of place fields for the proper place cells. (E) Mean size of place fields for the proper place cells. (F) Mean population sparseness. (G) Ratio of cells for which Hebbian learning of place fields was successful (according to the three similarity criteria defined in the Materials and Methods section). Parameter used as before 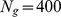, 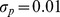 m, 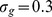, 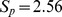, 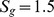, 4 modules, 15 realizations.

To test whether these observations are specific to the one-dimensional paradigm, we repeated the same simulations and analysis for a two-dimensional enclosure (see Materials and Methods and Fig. 2). As in the one-dimensional case, inspection of single examples for high numbers 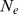 of remappings reveals that the place-selectivity of the readout neurons (the putative place cells) deteriorates much faster than the decoding quality (Fig. 7). Even random spatial patches (for 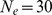; Fig. 7 B) allow for almost perfect decoding (Fig. 7 E). Spatial estimation only breaks down, if hardly any space modulation is observable in the firing patterns (Fig. 7 C, F). These exemplary observations are corroborated by a systematic quantitative assessment of the code and the firing fields in Fig. 8.

In analogy to the one-dimensional case, decoding quality increases with the number 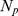 of putative place cells and remains in the centimeter range for 40 and more remappings if 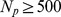 (Fig. 8A). At the same time, the place field characteristics deteriorate with increasing 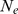 as was described in the one-dimensional case (Fig. 6): sparseness decreases (Fig. 8B, F), place field number increases before no clear place fields are visible anymore (Fig. 8C, D, E), place fields loose their similarity to the trained patterns (Fig. 8G).

In the two-dimensional case for few place cells 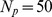, we observe an improvement in resolution when going from one to about 10 remappings before the decoding error again increases with 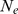. Although counter-intuitive, this effect reflects that an increase in mean population sparseness at first provides a better coverage of the square box. To make the model work also for small 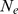, the number 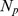 of place cells has to be large to overcome this finite size effect. It therefore imposes a constraint on a minimum number of 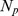. This effect also exemplifies that decoding RMSE depends on many different aspects and thus it is generally difficult to use it as a single measure for comparing the "quality" of a population code.

We also assessed the robustness of our findings with respect to essential model parameters. We evaluated the place code for different number of grid cells 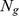, while keeping a constant total number 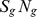 of input spikes and found essentially no difference (S1 Figure). Also, a mere increase in the number 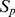 of place field spikes only improves the spatial resolution but does not alter any of the other place field characteristics (S2 Figure).

### Direct control of sparseness

**Figure 9:**
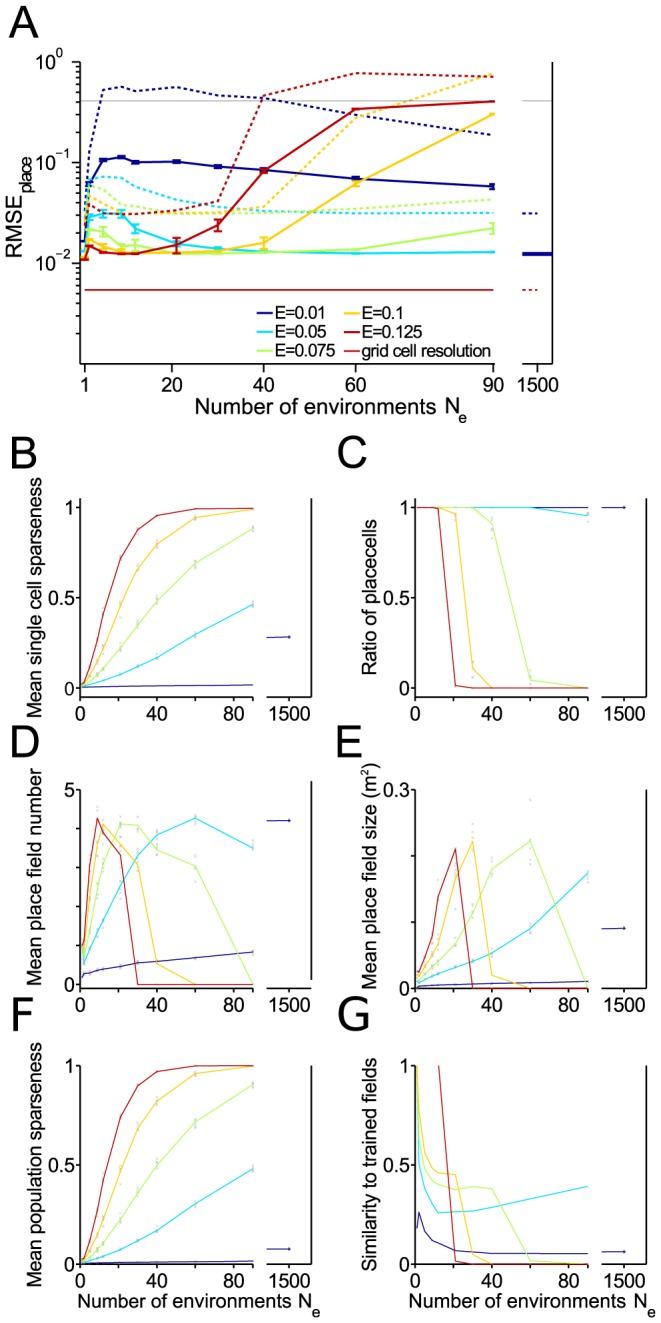
Effect of the E% parameter on the capacity for storing remappings in a square box for 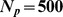 place cells. Place cell resolution and further measures as functions of the number 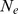 of remappings stored. (A) Root mean square error (RMSE) of place cells. Blue and green solid lines: Mean over realizations. Dashed lines: 99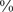 quantiles. Red line RMSE of the grid cell input. (B) Mean single cell sparseness. (C) Ratio of proper place cells. (D) Mean number of place fields for the proper place cells. (E) Mean size of place fields for the proper place cells. (F) Mean population sparseness. (G) Ratio of cells for which Hebbian learning of place fields was successful (according to the three similarity criteria defined in the Materials and Methods section). Parameter used as before 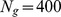, 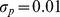 m, 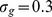, 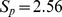, 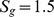, 4 modules, 8 realizations. The curve for 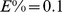 is taken from Figure 8 and has 15 realizations.

A substantial effect on the population code can be observed by altering the strength of feedback inhibition in the place field population by means of the E% value (Fig. 9). This parameter determines the firing threshold as the input strength E% below the maximum (see Methods and [21]). The E% value directly controls the sparseness of the code (Fig. 9B–G). For low E% values (sparse codes) and low numbers 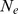 of environments, we again observe the finite size effect of high RMSE, which then improves with increasing 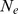 (Fig. 9A). This initially high RMSE, however, can again be compensated for by using larger numbers 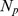 of place cells (as in Fig. 8 A). As a result, the decreasing E% generally allows to store more environments, however, at the cost of high 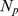 to achieve a sufficiently small RMSE for low 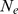.

### Partial learning

**Figure 10:**
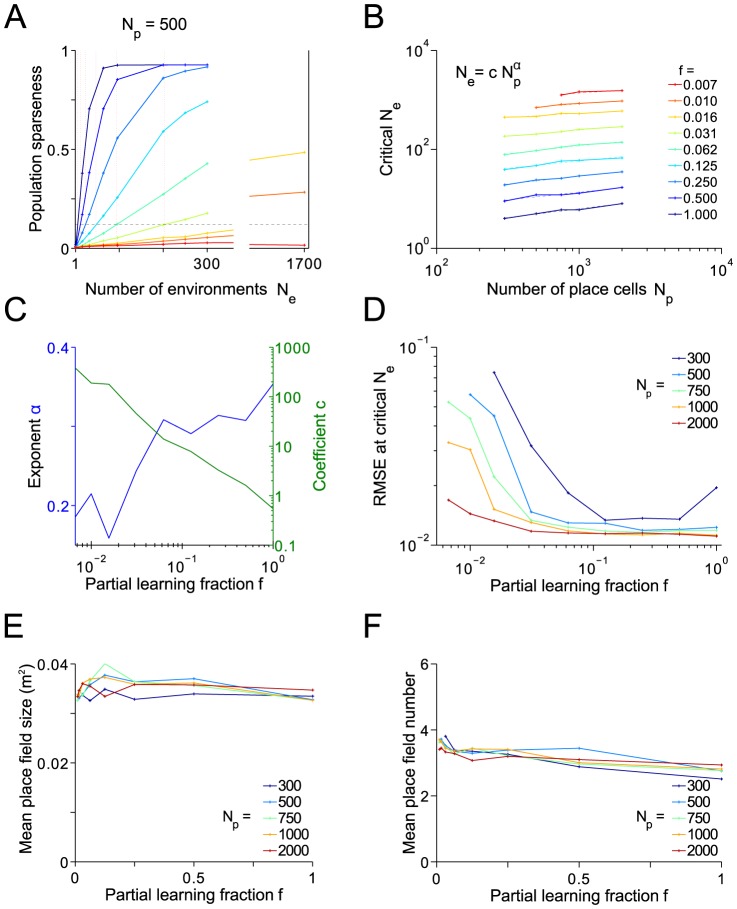
Partial learning. Effect of place cell number 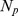 and of the fraction 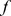 that are trained to encode one environment on the number of environments 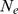. (A) Population sparseness as function of environments 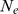 stored, for 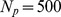 place cells. Different colors represent different fractions for partial learning, see legend in B. The critical value 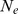 at which sparseness reaches a biologically realistic value of 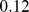 is obtained by interpolation. (B) Critical values of 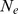 as function of place cell number 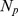 and partial learning fraction 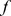. Data can be fitted by simple logarithmic functions 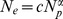. (C) Exponent 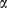 and coefficient 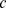 of fit from B. (D) Root mean square errors (RMSE) at the critical 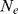 for the 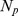 and 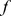 in B. (E) and (F): Mean place field size (E) and number (F) at the critical 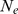 for the 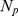 and 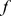 in B. Averages are over proper place cells.

If one constrains the parameter space to biologically realistic mean population sparseness values for the hippocampal place fields about 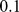 to 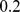 (Supporting Information of [24] and [25], see Discussion) our simulations of the standard parameter regime (Fig. 8) show that such a regular place code can only be observed for up to about ten environments. Also for increased E% value the number of sparsely encoded environments is only increased to several tens (Fig. 9). A major factor limiting the number 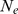 of environments is that in our model the synapses to the place cells are updated in each remapping, i.e., the place cells experience maximal interference. One can considerably extend the number of remappings for a given sparseness if the synaptic changes from different remappings are distributed to varying subsets of place cells, thereby increasing the overall number of putative place cells (partial learning). This strategy is motivated by an experimental report showing that only a small subset of CA1 pyramidal cells shows intracellular determinants for being recruited as a place cell in a novel environment [26]. We illustrate the benefits of partial learning by a further set of simulations in which the synaptic weights to only a fraction 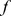 of the place cells are updated in each individual remapping (partial learning; Fig. 10). Using mean population sparseness as a criterion for the breakdown of the place code, partial learning increases the number of possible remappings (Fig. 10A) to over a hundred. As a measure for capacity, one can define a critical number of environments at which the mean population sparseness exceeds a (biologically motivated) threshold value of 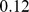 (see Discussion). This critical 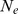 only weakly increases with the number 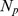 of place fields but strongly decreases with increasing fraction 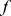 of partial learning (Fig. 10B, C).

In rat hippocampus the number 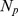 of CA1 neurons is in the order of several 100 thousands and thus according to Fig. 10B, a sparse place representation may still be consistent with storing hundreds to thousands of remappings if each place cell is involved in only a small fraction of environments.

The encoding acuity (RMSE) is generally not affected by partial learning as long as 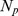 is not too small (Fig. 10D). Only for very small values of 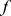, when a winner-take-all effect of the E%-MAX rule *decreases* sparseness for 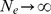, spatial acuity deteriorates. However, this regime is biologically unrealistic, since there the number 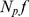 of neurons encoding an environment tends to zero.

The geometry of the spatial firing patterns (place field size and number), is virtually unaffected by 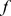 (Fig. 10 D, E). The place field sizes we find in the model (up to 0.05 m^2^) are within the range reported in the experimental literature [25], [27], the mean number of place fields (about 3) is at the upper bound of the 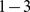 fields per m^2^ experimentally found in the hippocampus and dentate gyrus [24], [27], which indicates that the place code might in fact even be sparser than than the 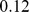 threshold motivated by current experimental data (see Discussion).

## Discussion

The hippocampal formation hosts two space representations. A sparse one in the hippocampus proper, in which the neurons have a limited number of distinct firing fields (place fields) and a dense one in the MEC, where grid cells exhibit multiple firing fields located on the nodes of a hexagonal lattice. If both brain regions encode the unique physical spatial position of the animal, the two codes have to be coherent. Anatomically both brain areas are reciprocally connected [5]–[8] and thus place cell activity will influence grid cell activity and vice versa.

In this paper, we focus on the connections from the medial entorhinal grid cells to the hippocampus, which anatomically correspond to the perforant pathway and the temporo-ammonic pathway. These pathways have initially been thought to predominantly underly the transformation from grid to place cells [19], [28]–[32]. More recently, developmental studies [12], [13] and pharmacological interventions that block grid cell firing [7], [14]–[16], have shown that place cells can also be observed independently of grid-field firing (but see [33]). Thus, while the MEC-to-hippocampus connections seem to be unnecessary to generate place fields, they are likely important in synchronizing both codes. This view is further corroborated by the observation that place cell firing is less stable if MEC input is eliminated [34].

Although it is known from information theory that capacity and sparseness cannot be maximized simultaneously [35], [36], our paper exemplifies this rule for a specific neuronal network example, in that it shows that maximization of capacity of MEC-to-hippocampal connections destroys the sparseness of the hippocampal place code.

From the theoretical perspective, if the synaptic matrix is know that transforms one code into another, reading out a dense code is more difficult than reading out a sparse code. This is because the synaptic matrix gives rise to a much noisier postsynaptic signal for dense input patterns [37]. Therefore the transformation from place cells to grid cells is less problematic than the other way round. The grid to place transformation provides an interesting test case to study information transfer between different brain areas in general.

Our model is largely based on experimental reports of grid and place cell remapping [18], [20], [38]–[40]. While place cells turn on, turn off, or show random relocation during global remapping [40], grid fields shift and rotate. In our model, we consider only shifts, since rotations were shown to be less efficient for remapping previously [19]. Although the grid modules seem to operate functionally independent [17], it is not yet clear whether the modules remap independently as proposed in [19]. A further finding from [19] was that a few (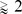) modules suffice for strong remapping and data [17] suggest that MEC has only about 5 to 9 modules. Only a part of these modules innervate any one place cell, owing to the dorso-ventrally ordered topography of the input fibers. We therefore concluded that a biologically reasonable number of modules influencing any single place cell is about 4. We further assume that the number of cells per module is constant, which is optimal from a theoretical perspective [9] but might not necessarily be the case [17].

To connect our simulations to hippocampal physiology, we assume a population sparseness value of 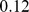. This value can be estimated by combining data from the supporting information (Table S1 of [24]) (mean number of place cells: 1.1/(0.8 m)^2^ for CA3, 2/(0.8 m)^2^ for DG; percentage of place fields: 62/71 for CA3, 41/44 for DG) and place field areas measured in [25] in a circular enclosure of diameter 76 cm (field area: 0.08 m^2^ for CA3, 0.06 m^2^ for DG). The estimate of the population sparseness for a 1 m^2^ enclosure (as in our simulations) thus follows from the product of these three values, i.e., we obtain about 0.12 for CA3 and 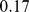 for DG. However, in our simulations, a sparseness value of 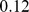 yields a number of place fields per place cell that is slightly higher than observed in experiments, and thus the above numbers may over-estimate the sparseness values in the real rodent brain.

Previous coding theories of MEC grid cells have extensively investigated spatial resolution. According to [9], [41], hierarchical grid codes outperform place codes by far in terms of their scaling behavior. A main reason is that for a constant resolution, the number of place cells scales with area, whereas for grid cells only those with larger period have to be scaled up with area for disambiguation, however, the resolution mostly rests on the smallest grid periodicity and thus the size of the population with small periodicity is independent of spatial range to be encoded. The parameter regimes in which grid codes are particularly superior to place codes provide relative root mean square errors in the range of 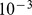 and even far below [9]. For a one meter environment, this would correspond to (sub-)millimeter resolution which is biologically irrelevant for encoding but might be important for MEC models of path integration [42], [43] where errors can accumulate over time. In the regime used for the present model (Figs. 3 and 4), the surplus in resolution of the grid code is relatively small, consistent with a biologically relevant decoding situation of high noise and few modules [44].

A further noteworthy result of our simulations is that a population code still contains almost maximal space information (in terms of minimal RMSE), even if no clear spatial firing fields can be delineated anymore. On the one hand this shows that also brain areas like the lateral entorhinal cortex [45] and the subiculum [46] with only weakly space-modulated individual neurons can provide high-resolution space information on the population level and thus a superposition of such weakly modulated firing fields via synaptic inputs is sufficient to provide place information to any downstream structure. This means that also the hippocampus and the MEC may not generate their strongly spatially modulated firing fields de-novo but inherit them from weakly modulated populations as e.g. the lateral entorhinal cortex. On the other hand our findings show that sparseness of the hippocampal place representation is not due to coding precision requirements but must serve other purposes. Manifold advantages of sparseness have been proposed [47] including energy efficiency [48]. A further classical benefit of sparse representations arises for auto-associative memory networks, where it facilitates memory retrieval due to reduced interference [37], [49]–[52].

Although our model includes lateral inhibition via the E% rule to limit the overall network activity the network cannot enforce sparseness except for unrealistically low values of 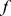. So it is still possible that other assumptions about the recurrent connections may enforce sparseness more effectively, while allowing remappings. For example, in a model using a combination of recurrent excitation and inhibition [53], [54] place fields arise from stable attractor states, where each attractor reflects the topology of place field positions for one remapping. The capacity (number of remappings per neuron) of this autoassociator is in the range of few percent and, thus for 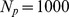 may end up slightly above the capacity derived from our model (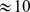) (for fixed realistic sparseness). So, recurrent excitatory connections between place cells can potentially help to keep the place fields compact. The disadvantage of attractor-like solutions is that they show catastrophic forgetting, whereas our model exhibits a gradual decline of the order parameters (Figs. 6, 8 and 9).

The view on how space information is communicated between the reciprocally connected brain areas hippocampus and MEC has recently undergone a dramatic change from a completely feed-forward grid-to-place dogma [19], [28]–[32] to an almost reversed place-to-grid picture [7], [12]–[16]. We started out under the assumption that the spatial precision in the hippocampus mostly relies on inputs from MEC grid cells and remapping the MEC triggers remapping on the hippocampus. If this was the only function of the MEC-to-hippocampus connections, they should be filled with as much space information as possible and the representation would no longer be sparse. Our results thus show that functionally the classical pure grid-to-place hypothesis would only suboptimally use the coding resources. The required compact place fields and the MEC-to-hippocampus synapses thus do not seem to be optimized to transfer space information.

Since new experimental data [7], [12]–[16] show that MEC is actually not essential for generating place cells, our findings suggest the possibility that hippocampal space information might actually primarily stem from other regions than the MEC. The grid field input to place fields thus likely imposes only modulatory or stabilizing effects. Conversely, no grid cells have been so far observed without place cell activity, and thus the place-to-grid hypothesis is still a possible candidate. However, it is unclear why hexagonal symmetry might emerge from the perspective of a transformation of a sparse place code to a dense code, and thus it might as well be that the two codes are generated independently for different computational purposes and the reciprocal connections are only required for synchronization and stabilization.

## Materials and Methods

### Grid cell firing rate maps in one dimension

The 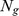 grid cells are modeled as Poisson spikers with firing maps 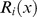 that denote the mean spike count of cell 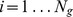 conditioned on the position 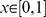 on a 1 meter track. All cells have the same maximal spike count 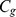 and the same field width parameter 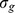. The cells differ in their spatial periods 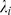 and grid phases 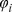. The specific model for the cells' Poisson spike counts follows a von Mises function:
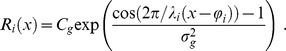



Each cell belongs to one of 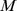 modules. Cells in a module share a spatial period 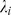. The phases 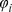 in each module are chosen equidistantly such that the firing fields cover the linear track; Fig. 1A.

Though we have only one width parameter 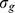 for all cells, the tuning width 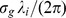 for the cells in one specific module scales with the period 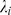, as can be seen from expanding the cosine term in 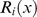.

The spike count 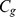 is adjusted such that the whole grid cell population generates a constant given number 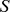 of spikes averaged over all positions 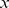 and cells 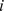, i.e.,
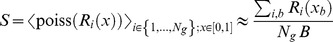
(1)


Here, the locations 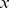 are discretized in 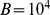 bins 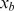. The value used for 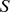 is 1.5 spikes per cell. Since for Poisson spikers the spike count is a product of averaging duration, firing rate and number of cells with the same rate function 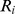, the three factors cannot be distinguished. Although, for simplicity, we call 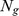 the number of grid cells, it is more correctly referred to as the number of grid cell channels (different rate functions 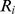).

The different modules are defined by their grid period 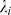. In our grid cell population, the first module is assigned the largest spatial period, which we take 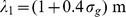 such that each cell in this module only has one unique firing field on the track. The smaller periods of the other modules are obtained via geometric progression, 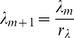, with a period ratio 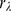, and 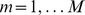. The period ratio 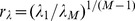 is defined via the number 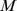 of modules and the smallest period 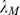, which is set to 30 cm, a lower bound suggested by experiments [3], [17]. Thus the only remaining degrees of freedom for the grid code are the number 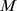 of modules, the width constant 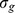 and the mean spike count per length 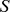. We choose 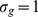, 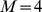 and 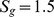 unless otherwise mentioned.

### Hebbian learning of place cells

The synaptic weights 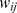 of the feed forward connections from grid to place cells are set by Hebbian learning based on the rate maps 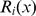 of the grid cells from eq. (1) and the desired rate maps
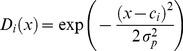
(2)of the place cells with width 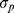 and centers 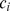 that uniformly cover the interval 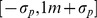; Fig. 1C.

With these idealized place fields, the weights are calculated according to outer product (Hebbian) rule: using discretized locations 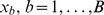 we define
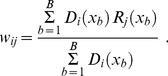
(3)


The denominator ensures that connections to place cells with fields at the borders are as strong as the ones to centered place fields.

### Remapping

The two networks (grid and place cells) are supposed to encode 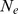 environments. Each environment has a new grid code generated by shifting each module's phases by a constant 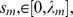 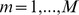. These shifts have experimentally been shown to be coherent within one module [18] and have been theoretically proposed to be uncorrelated between modules [19]. The shifted grid field patterns are denoted by 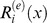. A new place code 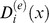 is generated by randomly choosing the place field centers 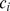. Hebbian learning as in eq. 3 is repeated 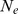 times and weights are added.

### Place cell spikes and position decoding

The place cell spikes for cell 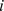 at a position 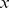 are produced by drawing Poisson spikes 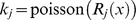 for the grid cells, then taking the weighted sum
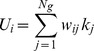
of those, to yield a membrane potential of the place cells. The activity is then generated following the E%-MAX rule [21], that emulates the effect of recurrent inhibition: after finding the maximum membrane potential 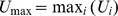, all 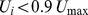 are set to zero and the ones above this threshold are multiplied with a constant 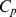, and used as place cell firing rate from which spike counts 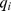 are derived according to Poisson statistics.

Decoding the place code via a minimum mean square estimator [55]

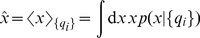
(4)requires a statistical model 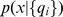 of place cell firing. Since in the model the single trial spike counts 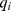 are statistically independent the posterior can be obtained using Bayes' rule,
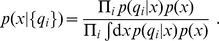



The prior is taken as constant, 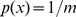. The individual likelihoods 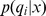 are obtained by repeating the above stochastic process 800 times for each cell and each sampled position and sampling the relative frequencies of spike counts 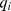. This distribution is then fitted with a bimodal model function consisting of a probability 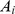 of cell 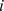 not firing, and probability of firing 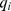 spikes following a normal distribution with fit parameters mean 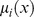 and variance 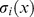:
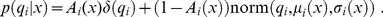
(5)


**Figure 11:**
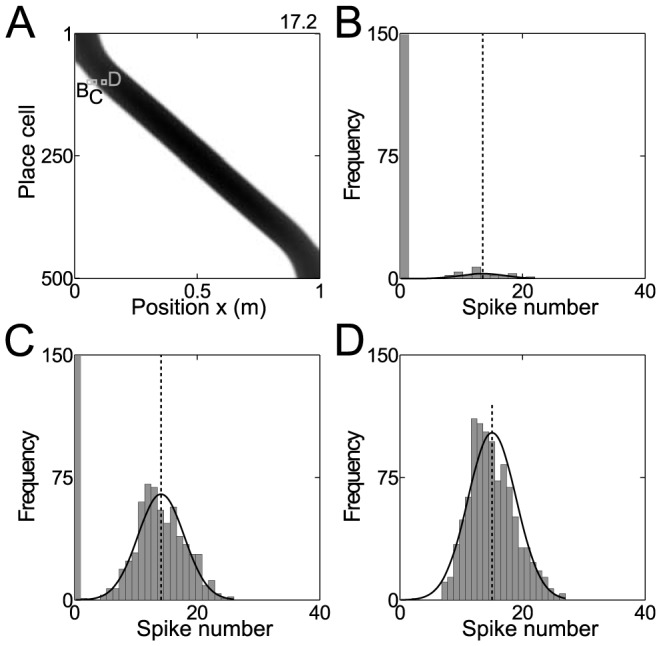
Spike count likelihood of place cells. (A) Firing rates (gray code) of model place cells as a function of position. (B–D) Simulated spike counts and fits of the model function eq. (5) for examples indicated in A.

Examples for such fits are shown in Fig. 11. Again, the constant 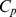 is obtained by fixing the number 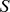 of spikes per centimeter per cell in an iterative fashion. The resulting value is 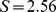 unless otherwise mentioned.

### Two-dimensional place code

For comparison we also implemented the model in two spatial dimensions 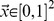. There, the grid cell's firing maps are set as in [31]

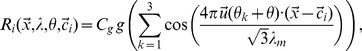
with 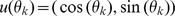 being a unitary vector pointing into direction 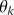. Using 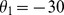, 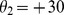 and 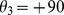, the three spatial waves add up to a hexagonal firing pattern with spatial period 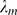, a maximum at 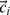, and orientation 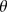 (Fig. 2A). The nonlinearity 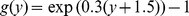 both adjusts the minimal firing rate to zero and matches the spatial decay of the firing rate peaks to experiments [31]. Like for the one-dimensional simulations we use four modules. Cells in one module share spatial period and orientation. The period for the first module is 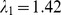 m (larger than the box). The smallest period is set to 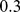 m. The two intermediate periods are again obtained by geometric progression 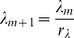. Orientation 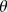 for each module is drawn at random. The "centers" 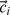 are uniformly distributed over the Wigner cell of size 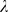. For all computational purposes, we used 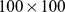 spatial bins to discretize the box.

To generate two-dimensional place fields we set feed-forward weights by Hebbian learning, using Gaussian tuning curves as firing maps for place fields as in eq. (2), but with 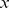 and 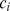 replaced by their two-dimensional counterparts (Fig. 2 B, C). The centers 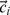 cover the box uniformly on a square grid. Centers of teacher place fields for cell exceeding the number of nodes on the square lattice were distributed randomly. Weights are then calculated using eq. (3).

The spikes are produced as in the one-dimensional case. Decoding follows eq. (4) with one-dimensional quantities replaced by their two-dimensional counterparts.

For a remapping, each grid cell module is assigned one random spatial shift vector, added to all 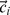 from that module. The shift is obtained by drawing a vector from the Wigner cell of that module using a uniform distribution (Fig. 2 D). For remapping, the place cells are assigned new centers at random, which again cover the box equidistantly. Then Hebbian learning is repeated, adding to the existing weights (Fig. 2 E, F).

### Partial learning

Partial learning as used in the simulations of Fig. 10 was implemented as follows. For each environment we selected a random set of 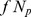 cells such that each cell is selected approximately the same amount of times across environments. This was achieved via random permutations of the cell indices. The sets of 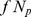 cells were taken from such a random index sequence one after the other, and only if less than 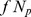 items were left in the index sequence, a new random permutation was generated.

For each set of 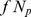 selected cells we defined teacher place fields that cover the whole environment as uniformly as possible on a square grid with 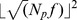 nodes (see previous section). Hebbian learning according to eq. (3) was applied to only the synapses between the grid field population and the selected set of postsynaptic cells.

By construction, some place cells will be used in more environments than others. We normalize the rows of 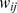 after all environments have been learned to avoid that the cells that are involved in more environments (and thus have larger weights) are overly excited and exert too much inhibition on the remaining cells via the E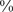-MAX rule.

### Single cell sparseness

According to [23], single cell sparseness is defined as 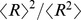, where 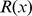 denotes the firing rate of the specific cell as a function of position 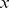 and 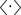 indicates the average over space.

### Population sparseness

Population sparseness is defined as the percentage of place cells firing above a threshold of 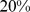 of the maximum firing rate at any position.

### Detection of (proper) place fields

The number and size of place fields was found by first thresholding the rate maps, discarding all bins below 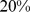 of the maximal rate, and then applying the algorithm by Hoshen and Kopelman [56]. Bins were considered neighboring if they share an edge, hence diagonal bins were not neighbors. Place fields were only included in the analysis (proper place fields) if they were larger than 50 cm^2^ and smaller than 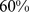 of the total environment.

### Success of Hebbian learning by similarity

Learning of place fields was considered successful in a cell if the learned field showed sufficient similarity to the training field according to three criteria: 1) the total area above a threshold of 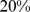 peak rate has to be smaller than 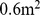, 2) the place field center has to be detected close to the desired location, i.e., no further away than the place field radius (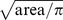), and 3) the desired place field has to have an area at least twice the size of all other place fields.

## Supporting Information

S1 FigureEffect of the place cell spike number 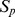 on the capacity for storing remappings in a square box. Place cell resolution and further measures as functions of the number 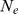 of remappings stored for 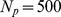. (A) Root mean square error (RMSE) of place cells. Blue and green solid lines: Mean over realizations. Dashed lines: 99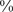 quantiles. Red line RMSE of the grid cell input. (B) Mean single cell sparseness. (C) Ratio of proper place cells. (D) Mean number of place fields for the proper place cells. (E) Mean size of place fields for the proper place cells. (F) Mean population sparseness. (G) Ratio of cells for which Hebbian learning of place fields was successful (according to the three similarity criteria defined in the Materials and Methods section). Parameter used as before 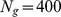, 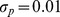 m, 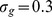, 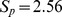, 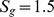, 4 modules, 15 realizations, 10 for 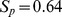.(EPS)

S2 FigureEffect of varying grid cell number 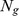 and grid cell spike count 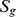 with constant 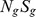 on the capacity for storing remappings in a square box. Place cell resolution and further measures as functions of the number 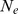 of remappings stored for 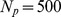. (A) Root mean square error (RMSE) of place cells. Blue and green solid lines: Mean over realizations. Dashed lines: 99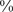 quantiles. Red line RMSE of the grid cell input. (B) Mean single cell sparseness. (C) Ratio of proper place cells. (D) Mean number of place fields for the proper place cells. (E) Mean size of place fields for the proper place cells. (F) Mean population sparseness. (G) Ratio of cells for which Hebbian learning of place fields was successful (according to the three similarity criteria defined in the Materials and Methods section). Parameters used are as before 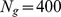, 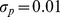 m, 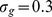, 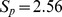, 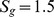, 4 modules, 7 realizations, 15 for 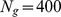, 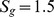, data from Fig. 8.(EPS)

## References

[1] O'Keefe J, Nadel L (1978) The Hippocampus as a Cognitive Map. Oxford University Press.

[2] Fyhn M, Molden S, Witter MP, Moser EI, Moser MB (2004) Spatial representation in the entorhinal cortex. Science 305: 1258–1264. 15333832 10.1126/science.1099901

[3] Hafting T, Fyhn M, Molden S, Moser MB, Moser EI (2005) Microstructure of a spatial map in the entorhinal cortex. Nature 436: 801–806. 15965463 10.1038/nature03721

[4] Hartley T, Lever C, Burgess N, O'Keefe J (2014) Space in the brain: how the hippocampal formation supports spatial cognition. Philos Trans R Soc Lond, B, Biol Sci 369: 20120510. 24366125 10.1098/rstb.2012.0510 PMC3866435

[5] Canto CB, Wouterlood FG, Witter MP (2008) What does the anatomical organization of the entorhinal cortex tell us? Neural Plast 2008: 381243. 18769556 10.1155/2008/381243 PMC2526269

[6] Zhang SJ, Ye J, Couey JJ, Witter M, Moser EI, et al (2014) Functional connectivity of the entorhinal-hippocampal space circuit. Philos Trans R Soc Lond, B, Biol Sci 369: 20120516. 24366130 10.1098/rstb.2012.0516 PMC3866440

[7] Bonnevie T, Dunn B, Fyhn M, Hafting T, Derdikman D, et al (2013) Grid cells require excitatory drive from the hippocampus. Nat Neurosci 16: 309–317. 23334581 10.1038/nn.3311

[8] Zhang SJ, Ye J, Miao C, Tsao A, Cerniauskas I, et al (2013) Optogenetic dissection of entorhinal-hippocampal functional connectivity. Science 340: 1232627. 23559255 10.1126/science.1232627

[9] Mathis A, Herz AV, Stemmler M (2012) Optimal population codes for space: grid cells outperform place cells. Neural Comput 24: 2280–2317. 22594833 10.1162/NECO_a_00319

[10] Fiete IR, Burak Y, Brookings T (2008) What grid cells convey about rat location. J Neurosci 28: 6858–6871. 18596161 10.1523/JNEUROSCI.5684-07.2008 PMC6670990

[11] Kubie JL, Muller RU (1991) Multiple representations in the hippocampus. Hippocampus 1: 240–242. 1669297 10.1002/hipo.450010305

[12] Wills TJ, Cacucci F, Burgess N, O'Keefe J (2010) Development of the hippocampal cognitive map in preweanling rats. Science 328: 1573–1576. 20558720 10.1126/science.1188224 PMC3543985

[13] Langston RF, Ainge JA, Couey JJ, Canto CB, Bjerknes TL, et al (2010) Development of the spatial representation system in the rat. Science 328: 1576–1580. 20558721 10.1126/science.1188210

[14] Koenig J, Linder AN, Leutgeb JK, Leutgeb S (2011) The spatial periodicity of grid cells is not sustained during reduced theta oscillations. Science 332: 592–595. 21527713 10.1126/science.1201685

[15] Brandon MP, Bogaard AR, Libby CP, Connerney MA, Gupta K, et al (2011) Reduction of theta rhythm dissociates grid cell spatial periodicity from directional tuning. Science 332: 595–599. 21527714 10.1126/science.1201652 PMC3252766

[16] Brandon MP, Koenig J, Leutgeb JK, Leutgeb S (2014) New and Distinct Hippocampal Place Codes Are Generated in a New Environment during Septal Inactivation. Neuron 82: 789–796. 24853939 10.1016/j.neuron.2014.04.013 PMC4294702

[17] Stensola H, Stensola T, Solstad T, Froland K, Moser MB, et al (2012) The entorhinal grid map is discretized. Nature 492: 72–78. 23222610 10.1038/nature11649

[18] Fyhn M, Hafting T, Treves A, Moser MB, Moser EI (2007) Hippocampal remapping and grid realignment in entorhinal cortex. Nature 446: 190–194. 17322902 10.1038/nature05601

[19] Monaco JD, Abbott LF, Abbott LF (2011) Modular realignment of entorhinal grid cell activity as a basis for hippocampal remapping. J Neurosci 31: 9414–9425. 21697391 10.1523/JNEUROSCI.1433-11.2011 PMC3143841

[20] Leutgeb S, Leutgeb JK, Barnes CA, Moser EI, McNaughton BL, et al (2005) Independent codes for spatial and episodic memory in hippocampal neuronal ensembles. Science 309: 619–623. 16040709 10.1126/science.1114037

[21] de Almeida L, Idiart M, Lisman JE (2009) A second function of gamma frequency oscillations: an E%-max winner-take-all mechanism selects which cells fire. J Neurosci 29: 7497–7503. 19515917 10.1523/JNEUROSCI.6044-08.2009 PMC2758634

[22] Bethge M, Rotermund D, Pawelzik K (2002) Optimal short-term population coding: when Fisher information fails. Neural Comput 14: 2317–2351. 12396565 10.1162/08997660260293247

[23] Treves A, Rolls ET (1992) Computational constraints suggest the need for two distinct input systems to the hippocampal CA3 network. Hippocampus 2: 189–199. 1308182 10.1002/hipo.450020209

[24] Leutgeb JK, Leutgeb S, Moser MB, Moser EI (2007) Pattern separation in the dentate gyrus and CA3 of the hippocampus. Science 315: 961–966. 17303747 10.1126/science.1135801

[25] Park E, Dvorak D, Fenton AA (2011) Ensemble place codes in hippocampus: CA1, CA3, and dentate gyrus place cells have multiple place fields in large environments. PLoS ONE 6: e22349. 21789250 10.1371/journal.pone.0022349 PMC3137630

[26] Epsztein J, Brecht M, Lee AK (2011) Intracellular determinants of hippocampal CA1 place and silent cell activity in a novel environment. Neuron 70: 109–120. 21482360 10.1016/j.neuron.2011.03.006 PMC3221010

[27] Jung MW, McNaughton BL (1993) Spatial selectivity of unit activity in the hippocampal granular layer. Hippocampus 3: 165–182. 8353604 10.1002/hipo.450030209

[28] Solstad T, Moser EI, Einevoll GT (2006) From grid cells to place cells: a mathematical model. Hippocampus 16: 1026–1031. 17094145 10.1002/hipo.20244

[29] Blair HT, Welday AC, Zhang K (2007) Scale-invariant memory representations emerge from moir interference between grid fields that produce theta oscillations: a computational model. J Neurosci 27: 3211–3229. 17376982 10.1523/JNEUROSCI.4724-06.2007 PMC6672484

[30] Molter C, Yamaguchi Y (2008) Impact of temporal coding of presynaptic entorhinal cortex grid cells on the formation of hippocampal place fields. Neural Netw 21: 303–310. 18242058 10.1016/j.neunet.2007.12.032

[31] de Almeida L, Idiart M, Lisman JE (2009) The input-output transformation of the hippocampal granule cells: from grid cells to place fields. J Neurosci 29: 7504–7512. 19515918 10.1523/JNEUROSCI.6048-08.2009 PMC2747669

[32] Cheng S, Frank LM (2011) The structure of networks that produce the transformation from grid cells to place cells. Neuroscience 197: 293–306. 21963867 10.1016/j.neuroscience.2011.09.002 PMC3210383

[33] Azizi AH, Schieferstein N, Cheng S (2014) The transformation from grid cells to place cells is robust to noise in the grid pattern. Hippocampus 24: 912–919. 24866281 10.1002/hipo.22306

[34] Schlesiger MI, Cannova CC, Mankin EA, Boublil BB, Hales JB, et al. (2013) The medial entorhinal cortex is required for hippocampal phase precession. In: Society for Neuroscience Meeting. 578.29/KKK68.

[35] Cover T, A TJ (1991) Elements of information theory. New York: Wiley.

[36] Rolls E, Treves A (1998) Neural Networks and Brain Function. Oxford: Oxford University Press.

[37] Willshaw DJ, Buneman OP, Longuet-Higgins HC (1969) Non-holographic associative memory. Nature 222: 960–962. 5789326 10.1038/222960a0

[38] Muller RU, Kubie JL (1986) The effects of changes in the environment on the spatial firing of hippocampal complex-spike cells. J Neurosci 7: 1951–1968. 3612226 10.1523/JNEUROSCI.07-07-01951.1987 PMC6568940

[39] Bostock E, Muller RU, Kubie JL (1991) Experience-dependent modifications of hippocampal place cell firing. Hippocampus 1: 193–205. 1669293 10.1002/hipo.450010207

[40] Leutgeb S, Leutgeb JK, Treves A, Moser MB, Moser EI (2004) Distinct ensemble codes in hippocampal areas CA3 and CA1. Science 305: 1295–1298. 15272123 10.1126/science.1100265

[41] Mathis A, Herz AV, Stemmler MB (2012) Resolution of nested neuronal representations can be exponential in the number of neurons. Phys Rev Lett 109: 018103. 23031134 10.1103/PhysRevLett.109.018103

[42] Fuhs MC, Touretzky DS (2006) A spin glass model of path integration in rat medial entorhinal cortex. J Neurosci 26: 4266–4276. 16624947 10.1523/JNEUROSCI.4353-05.2006 PMC6674007

[43] Burgess N, Barry C, O'Keefe J (2007) An oscillatory interference model of grid cell firing. Hippocampus 17: 801–812. 17598147 10.1002/hipo.20327 PMC2678278

[44] Mathis A, Herz AV, Stemmler MB (2013) Multiscale codes in the nervous system: the problem of noise correlations and the ambiguity of periodic scales. Phys Rev E Stat Nonlin Soft Matter Phys 88: 022713. 24032870 10.1103/PhysRevE.88.022713

[45] Hargreaves EL, Rao G, Lee I, Knierim JJ (2005) Major dissociation between medial and lateral entorhinal input to dorsal hippocampus. Science 308: 1792–1794. 15961670 10.1126/science.1110449

[46] Kim SM, Ganguli S, Frank LM (2012) Spatial information outflow from the hippocampal circuit: distributed spatial coding and phase precession in the subiculum. J Neurosci 32: 11539–11558. 22915100 10.1523/JNEUROSCI.5942-11.2012 PMC3458125

[47] Olshausen BA, Field DJ (2004) Sparse coding of sensory inputs. Curr Opin Neurobiol 14: 481–487. 15321069 10.1016/j.conb.2004.07.007

[48] Levy WB, Baxter RA (1996) Energy efficient neural codes. Neural Comput 8: 531–543. 8868566 10.1162/neco.1996.8.3.531

[49] Tsodyks M, Feigel'man M (1988) Enhanced storage capacity in neural networks with low level of activity. Europhys Lett 6: 101–105.

[50] Nadal JP (1991) Associative memory: on the (puzzling) sparse coding limit. J Phys A 24: 1093–1101.

[51] Leibold C, Kempter R (2006) Memory capacity for sequences in a recurrent network with biological constraints. Neural Comput 18: 904–941. 16494695 10.1162/089976606775774714

[52] Kammerer A, Tejero-Cantero A, Leibold C (2013) Inhibition enhances memory capacity: optimal feedback, transient replay and oscillations. J Comput Neurosci 34: 125–136. 22782801 10.1007/s10827-012-0410-z

[53] Monasson R, Rosay S (2013) Crosstalk and transitions between multiple spatial maps in an attractor neural network model of the hippocampus: Phase diagram. Phys Rev E 87: 062813. 10.1103/PhysRevE.87.062813 23848735

[54] Monasson R, Rosay S (2014) Crosstalk and transitions between multiple spatial maps in an attractor neural network model of the hippocampus: Collective motion of the activity. Phys Rev E 89: 032803. 10.1103/PhysRevE.89.032803 24730895

[55] Lehmann E, Casella C (1998) Theory of Point Estimation. Springer, New York.

[56] Hoshen J, Kopelman R (1976) Percolation and cluster distribution. I. cluster multiple labeling technique and critical concentration algorithm. Phys Rev B 14: 3438–3445.

